# Prognostic significance of KLF4 in solid tumours: an updated meta-analysis

**DOI:** 10.1186/s12885-022-09198-9

**Published:** 2022-02-17

**Authors:** Xiaoya Luo, Yue Zhang, Ying Meng, Ming Ji, Yongjun Wang

**Affiliations:** 1 Department of Gastroenterology, Beijing Friendship Hospital, Capital Medical University. National Clinical Research Center for Digestive Disease. Beijing Digestive Disease Center. Beijing Key Laboratory for Precancerous Lesion of Digestive Disease., Beijing, 100050 China; 2 Department of Oncology, The First Hospital of Fangshan District, Beijing, 102400 China

**Keywords:** KLF4, Solid tumours, Prognosis

## Abstract

**Background:**

Kruppel-like factor 4 (KLF4) is a zinc finger-containing transcription factor predominantly expressed in terminally differentiated epithelial tissues. Many studies have shown that KLF4 has various mechanisms in different tumours; however, the prognostic role of KLF4 remains unclear.

**Methods and results:**

We searched the relevant literature that evaluated the prognostic value of KLF4 in different cancers, and the original survival data were obtained from the text, tables or Kaplan–Meier curves for both comparative groups. Thirty studies were included in this meta-analysis, and a total of 10 malignant tumours were involved. The expression of KLF4 was not associated with the prognosis for overall survival (hazard ratio(HR)0.86, 95% confidence interval (CI): 0.65–1.13, *P* = 0.28), disease-free survival/recurrence-free survival/metastasis-free survival (HR 0.87, 95% CI: 0.52–1.44, *P* = 0.58) or disease-specific survival (HR 1.13, 95% CI: 0.44–2.87, *P* = 0.8).

**Conclusion:**

This study showed that the expression of KLF4 was not related to the prognosis of the tumours that were included in the study.

## Background

Cancer is a major public health problem worldwide and a heavy burden on society worldwide. In 2021, 1,898,160 new cancer cases and 608,570 cancer deaths are projected to occur in the United States, and cancer is the second leading cause of death in the United States [1]. Despite the rapid progression in the aetiology, diagnosis and treatment of cancer, the prognosis of malignant tumours remains unsatisfactory. Currently, in an era of precision therapy, the widespread use of molecular-targeted therapy has not only improved the survival rate of malignancies but also offered promising prospects in the treatment of cancer. The key to targeted therapy is to choose the most appropriate patients; thus, researchers should try their best to identify the ideal molecular markers. Therefore, the markers should represent both therapeutic value and prognostic value, as well as help the risk stratification and optimal treatment options for patients.

Kruppel-like factor 4 (KLF4), a member of the KLF family, is a zinc finger-containing transcription factor predominantly expressed in terminally differentiated epithelial tissues, including skin, lung and gastrointestinal tract. There are three functional domains in KLF4: a carboxyl-terminal DNA-binding domain containing three C2H2 zinc fingers, an activated or suppressed domain and nuclear location sequences [2, 3]. KLF4 binds to specific DNA sequences, including CACCC boxes and GC boxes, and regulates cellular proliferation.

KLF4 plays important roles in development, cellular reprogramming and cancer. It was initially thought to be a negative regulator of cell growth, with the ability to regulate the expression of a number of genes involved in cell cycle progression [4, 5]. A variety of studies have indicated that KLF-4 is a context-dependent oncogene or tumour suppressor gene that is regulated in many molecular pathways and cellular processes. Some studies have shown that KLF4 expression is decreased in gastric cancers, hepatocellular carcinoma and lung cancer and is a favourable prognostic factor [6–9]. However, some studies have shown that KLF4 expression is associated with poor survival in breast cancer, prostate cancer, colorectal cancer, and skin squamous cell carcinoma, which indicates that KLF4 may be an oncogene [6, 10–12]. Additionally, some recent studies have shown that patients with higher KLF4 expression have better overall survival and disease-free survival rates in breast cancer and prostate cancer [13, 14]. Moreover, there was no association between the KLF4 expression and pathological diagnosis and tumour–node–metastasis (TNM) stage [15]. Although most of the studies on KLF4 have focused on epithelial tumours, several studies have investigated the role of KLF4 in B cells and B cell malignancies and indicated that it is a tumour suppressor in non-Hodgkin’s lymphoma and a potential biomarker for inferior overall survival [16, 17].

The prognostic role of KLF4 remains unclear. In this study, we conducted a systematic review and meta-analysis to summarize the global findings in using KLF4 for the prediction of the clinical results of cancer patients.

## Methods

### Search strategy

A thorough search was carried out for all relevant literature that evaluated the prognostic value of KLF4 in different cancers until December 1, 2019, among the following electronic databases: PubMed, ISI Web of Science and Embase. Search terms were as follows: (KLF4 OR Krüppel-like factor 4 OR Gutenriched KLF OR GKLF OR ZEF OR Epithelial Zinc Finger Protein) AND (cancer OR tumour OR neoplasm OR carcinoma) AND (Prognosis OR prognostic OR survival OR outcome). The Cochrane Library was also reviewed for related papers. In addition, the citation lists of identified articles were manually reviewed to complete the search. Two authors independently performed this procedure. Any disagreement was resolved by mutual discussion.

### Selection criteria

In this meta-analysis, the eligibility of candidate studies was determined based on the following criteria: (i) studied patients with all kinds of cancers; (ii) measured KLF4 expression using either semiquantitative immunohistochemistry (IHC) or quantitative reverse transcription PCR (RT-PCR); and (iii) evaluated the correlation between KLF4 expression and prognosis. Articles were not taken into account when the following criteria were met: (i) duplicated or overlapping studies; (ii) reviews, case reports, comments, or conference abstracts; and (iii) absence of key information for further quantification calculation. Two individuals separately carried out all evaluations, and any discrepancy was resolved by consensus.

### Data extraction and conversion

Data retrieved from the reports included the following elements: author, publication year, origin of population, tumour type, follow-up time, sample size, KLF4 measurement method, cut-off value of the hazard ratios (HRs), and 95% confidence intervals (CIs) of KLF4 for overall survival (OS), disease-free survival (DFS), recurrence-free survival (RFS), progression-free survival (PFS), and disease-specific survival (DSS). The original survival data were obtained from the text, tables or Kaplan-Meier curves for both comparative groups. Engauge Digitizer 4.1 (downloaded from http://markummitchell.github.io/engauge-digitizer) helped us digitize and extract survival information from the Kaplan-Meier curves using the method established by Tierney et al. [18]

### Statistical analysis

The HRs in combination with the corresponding 95% CIs of identified studies were combined to estimate the overall effective value following Tierney’s method. Cochran’s Q test and Higgin’s I^2^ statistics were simultaneously adopted to test the heterogeneity of combined HRs. A random effects model was adopted to aggregate the pooled HR when significant heterogeneity existed (*I*^2^ > 50%); in contrast, a fixed effects model was employed (*I*^2^ < 50%). The impact of decreased KLF4 expression on prognosis was measured by the combined HRs and their corresponding 95% CIs extracted from each included article. Indirect HRs with related 95% CIs were obtained via the method established by Tierney. Generally, a pooled HR of > 1 was assumed to indicate a significant association with poor prognosis and was interpreted as statistically significant when its 95% CI did not cross 1.

## Results

### Characteristics of the included studies

**Fig. 1:**
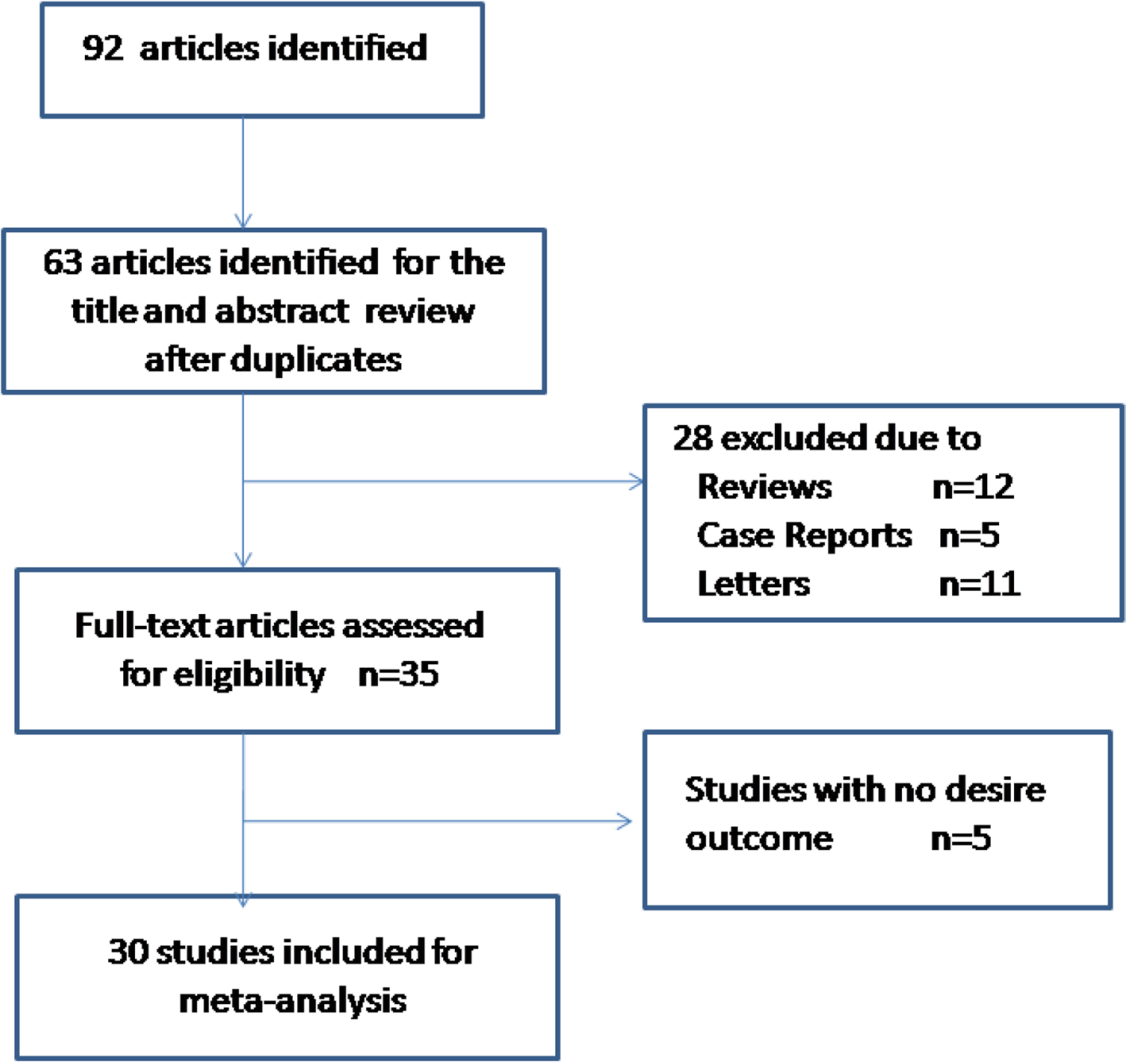
Flow chart of the search process

A flow diagram of the search process is given in Fig. 1. Ninety-two entries were identified from a primary literature search in PubMed, ISI Web of Science and Embase. Nineteen duplicates were removed. After manual screening of the titles and abstracts, we excluded 28 articles, such as basic studies, animal studies, noncancer subjects, non-KLF4 topics, or HRs or OS data that were unavailable. Thus, 30 studies [10, 19–45] were included in this meta-analysis. A total of 10 malignant tumours were involved.

Studies concerning hepatocellular carcinoma (HCC) are the largest group among all primary studies (*n* = 5), followed by colorectal cancer (CRC) (*n* = 4), breast cancer (BC) (*n* = 4), head and neck tumour (HNC) (*n* = 3), gastric cancer (GC) (n = 3), oesophageal squamous cell carcinoma (ESCC) (*n* = 3), pancreatic ductal adenocarcinoma (PDAC) (*n* = 3), urological cancer (*n* = 3), non-Hodgkin’s lymphoma (NHL) (*n* = 1), and cervical squamous cell carcinoma (CSCC) (*n* = 1). The majority of studies were carried out in China (*n* = 17), followed by Japan (*n* = 5) and other nations. The sample size of the identified articles ranged from 22 to 365, with a mean number of 128 patients. A total of 26 studies described the correlation of overall survival and KLF4 expression, one study reported only HR, and the other 25 studies reported both HR and 95% CI. The rest of the detailed features were recorded and summarized in Table 1.

**Table 1:** Characteristics of the included studies

Author, year	No.(M/F)	Tumor Location	Method	Stage	KLF4 +/−	Region	Anasysis	OS	DFS
								Hazard ratio	Hazard ratio
Zhen Liu 2013 [27]	165(112/53)	NPC	IHC	I-IV	63/102	China	OS	0.25	
Hongcheng Sun 2016 [23]	98(84/14)	HCC	IHC	I-III	NR	USA	OS/DFS	4.59	5.42
Qi Li 2012 [24]	40 (NR)	HCC	IHC		NR	China	OS	30.74	
Hui-Ting Hsu 2014 [37]	205(121/84)	HCC	IHC	I-IV	45/160	China(Taipei)	DFS		0.398
Xin Yin 2013 [19]	57(48/9)	HCC	PCR	I-III	7/50	China	OS/DFS	5.08	2.88
H Sun 2017 [30]	148(129/19)	HCC	IHC	I-III	81/67	China	OS/DFS	0.29	0.29
Heng Li 2013 [41]	149(106/43)	RCC	IHC	I-IV	103/46	China	OS/DFS	0.65	0.48
Heng Li 2014 [39]	139(102/37)	UCB	IHC	Ta-T1	56/83	China	RFS		0.51
Wei-Cheng Tseng 2016 [25]	227(146/81)	UCB	IHC	Ta-T4	73/154	China(Taipei)	OS	1.39	
Shyh-Kuan Tai 2011 [31]	62(58/4)	HNSCC	IHC	I-IV	20/42	China(Taipei)	DSS		2.13
Nilesh Patel 2010 [20]	365(192/173)	colon cancer	IHC	I-IV	249/106	USA	OS/DFS	0.87	0.62
Ha-young Lee 2014 [36]	125 (82/43)	CRC	PCR	I-IV	45/80	South Korea	OS	2.6	
Jing Xu 2008 [38]	60 (NR)	CRC	IHC	NR	18/42	China	OS	1.03	
W Tang 2014 [29]	85(46/39)	CRC	PCR	I-IV	43/42	China	OS	0.56	
Ming-Quan Ma 2014 [34]	98(64/34)	ESCC	IHC	I-III	43/55	China	OS	0.79	
Yutaka Shimada 2012 [28]	80 (71/9)	ESCC	IHC	I-IV	30/50	Japan	DSS	0.79	
Chang Yuan 2016 [22]	126(0/126)	BC	IHC	I-III	61/65	China	OS/DFS	0.67	0.53
Takuya Nagata 2016 [13]	208(0/208)	TNBC	IHC	I-III	100/108	Japan	OS/DFS	0.56	0.69
Takuya Nagata 2012 [15]	100(0/100)	BC	IHC	I-III	44/56	Japan	OS/DFS	0.318	0.256
Ashka Y 2004 [10]	146 (0/146)	BC	IHC	I-IV	114/32	Britain	OS	0.46	
Li-Sung Hsu 2013 [35]	118(82/36)	GC	IHC	I-IV	87/31	China	OS	0.65	
Daoyan Wei 2005 [43]	39(27/12)	GC	IHC	I-IV	12/27	USA	OS	2.18	
Isaya Hashimoto 2017 [40]	108(77/31)	GC	IHC	I-IV	72/36	Japan	OS	0.57	
Niccola Funel 2011 [33]	22 (NR)	PDAC	PCR	NR	6/16	Italy	OS/DFS	2.5	2.6
Zhulin Yang 2016 [26]	106(61/45)	PDAC	IHC	I-IV	47/59	China	OS	0.38	
Daoyan Wei 2010 [21]	22(NR)	PDAC	IHC	II	4/18	USA	OS	2.01	
Hai-Xia Liu 2017 [42]	117(0/117)	LACSCC	IHC	II-IV	53/64	China	OS/PFS	1.29	2.55
Rumi Yoshihama 2016 [32]	108(69/39)	OSCC	IHC	T1-T4	53/55	Japan	OS/DFS	1.17	1.65
Chih-Jung Chen 2011 [44]	215(205/10)	OSCC	IHC	I-IV	191/24	China(Taiwan)	OS	0.46	
Alberto Valencia-Hipólito 2014 [45]	73(46/27)	NHL	IHC	I-IV	48/25	Mexico	OS	1.84	

*CRC* colorectal cancer, *GC* gastric cancer, *HCC* hepatocellular carcinoma, *ESCC* esophageal squamous cell carcinoma, *PDAC* pancreatic ductal adenocarcinoma, *IHC* immunohistochemistry, *RT-PCR* reverse transcription polymerase chain reaction, *OS* overall survival, *DFS* disease-free survival, *DSS* disease-specific survival, *PFS* Progression-free survival, *NR* not report, *NPC* nasopharyngeal carcinoma, *BC* breast cancer, *RCC* renal cell carcinoma, *OSCC* Oral squamous cell carcinoma, *LACSCC* locally advanced cervical squamous cell carcinoma, *NHL* non-Hodgkin lymphomas, *HNSCC* head and neck squamous cell carcinoma, *SC* survival curve, *95% CI* 95% confidence interval, *HR* hazard ratio

### Meta-analysis

**Fig. 2:**
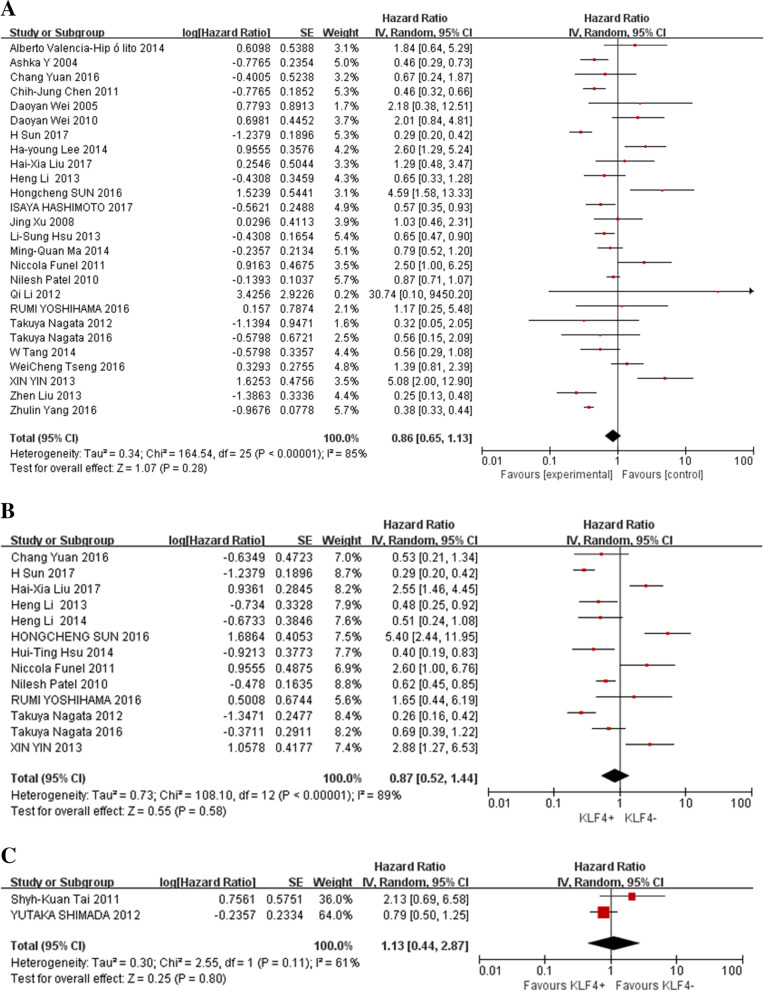
Forest plot and pooled HR and 95% CI for OS (**A**), PFS (**B**) and DSS(**C**)

**Fig. 3:**
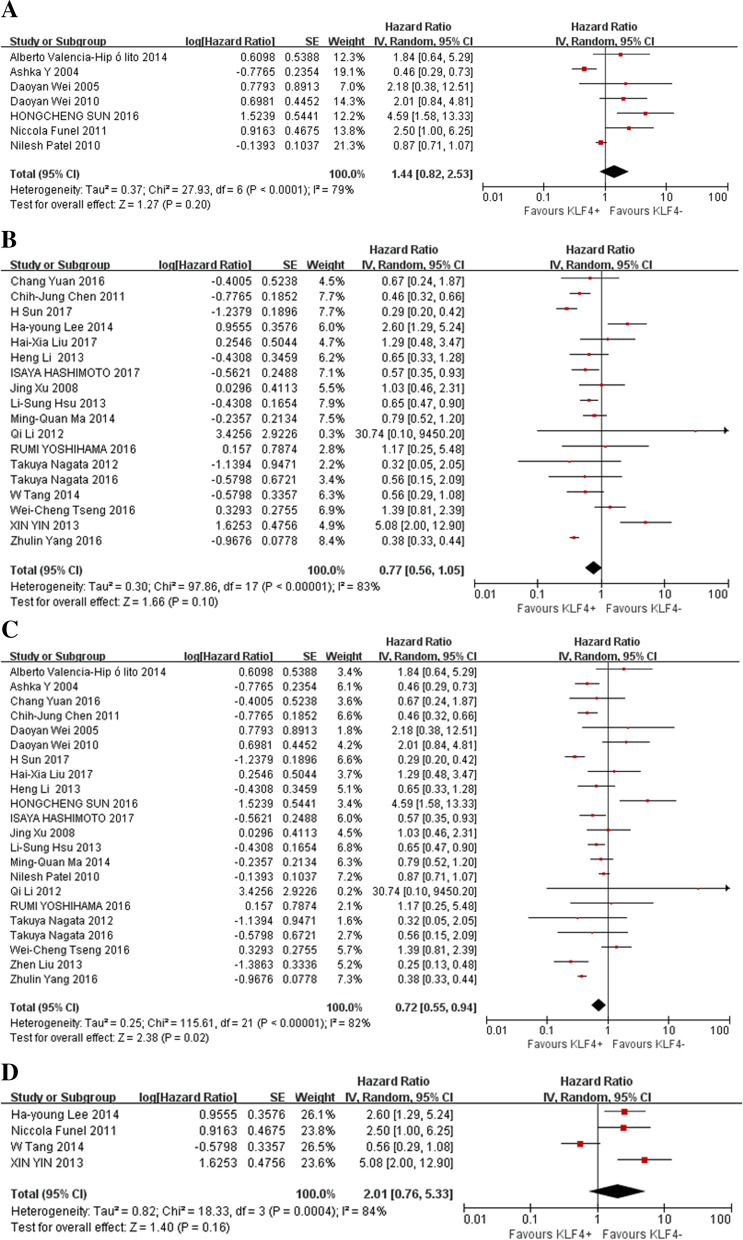
Forest plot and pooled HR and 95% CI for OS of the subgroup analysis (ethnicity and measurement method) (**A**: OS of subgroups of non-Asian patients; **B**: OS of subgroups of Asian patients; **C**: OS of subgroups of IHC; **D**: OS of subgroups of RT-PCR)

**Fig. 4:**
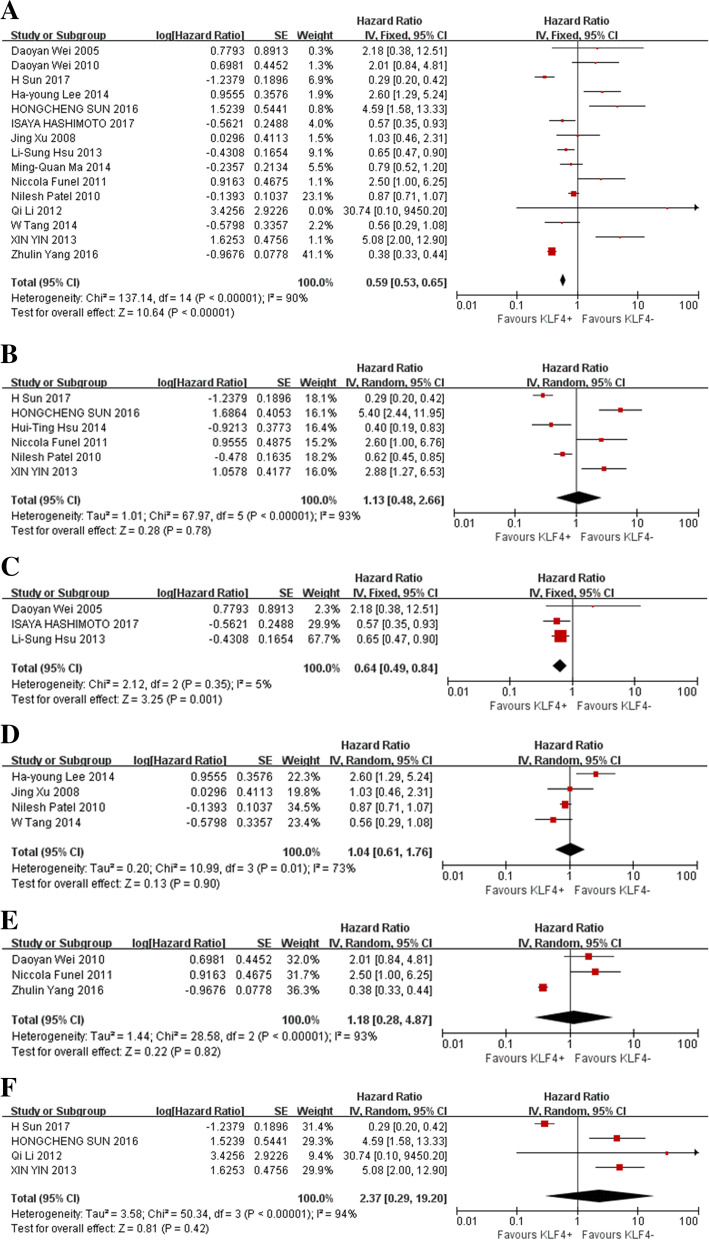
Forest plot and pooled HR and 95% CI for OS and DFS of digestive system cancers (**A**: OS of digestive system cancers; **B**: DFS of digestive system cancers; **C**: OS of subgroups of gastric cancer (GC); **D**: OS of subgroups of colorectal cancer (CRC); **E**: OS of subgroups of pancreatic ductal adenocarcinoma (PDAC); **F**: OS of subgroups of hepatocellular cancer (HCC))

The association between KLF4 expression and cancer prognosis is illustrated in Figs. 2, 3 and 4. Overall, the expression of KLF4 was not associated with the prognosis in those patients, with a pooled HR of 0.86 (95% CI: 0.65–1.13, *P* = 0.28) for OS via a random model because of the significant heterogeneity (I^2^ = 85%) (Fig. 2A). In the same way, the expression of KLF4 had nothing to do with DFS, RFS and metastasis-free survival (MFS), with a pooled HR of 0.87 (95% CI: 0.52–1.44, *P* = 0.58) calculated by a random model because of the presence of profound heterogeneity (I^2^ = 89%) (Fig. 2B). Additionally, KLF4 was not related to DSS, with a pooled HR of 1.13 (95% CI: 0.44–2.87, *P* = 0.8) through a random effects model for insignificant heterogeneity (I^2^ = 61%) (Fig. 2C).

To explore the sources of heterogeneity, subgroup analyses for OS were conducted by ethnicity, measurement method, and cancer type. In the ethnicity subgroup analyses, the results showed that KLF4 expression had no impact on OS (HR = 0.77, 95% CI: 0.56–1.05, *P* = 0.1) in Asian cancer patients (Fig. 3B) or OS in non-Asian patients (HR = 1.44, 95% CI: 0.82–2.53, *P* = 0.2) (Fig. 3A). In the subgroup analyses by the measurement methods, high expression of KLF4 in the IHC group showed improved OS (HR = 0.72, 95% CI: 0.55–0.94, *P* = 0.02) (Fig. 3C). In the RT-PCR group, KLF4 was not associated with OS (HR =2.01, 95% CI: 0.76–5.33, *P* = 0.16) (Fig. 3D).

In the stratified analyses according to cancer type, high KLF4 expression indicated better prognosis in patients with GC, HCC, PDAC and CRC, with a pooled HR of 0.59 (95% CI: 0.53–0.65, *P* < 0.001) for OS (Fig. 4A). Disappointedly, no PFS/DFS/RFS benefits were observed (HR = 1.13, 95% CI: 0.48–2.66, *P* = 0.78) (Fig. 4B). For different types of digestive system cancers, our research showed that high expression of KLF4 was associated with a significantly prolonged OS in GC (HR = 0.64, 95% CI: 0.49–0.84, *P* = 0.001) (Fig. 4C) but not statistically significant in CRC (HR = 1.04, 95% CI: 0.61–1.76) (Fig. 4D), PDAC (HR = 1.18, 95% CI: 0.28–4.87) (Fig. 4E) or HCC for OS (HR = 2.37, 95% CI: 0.29–19.20, *P* = 0.42) (Fig. 4F).

**Fig. 5:**
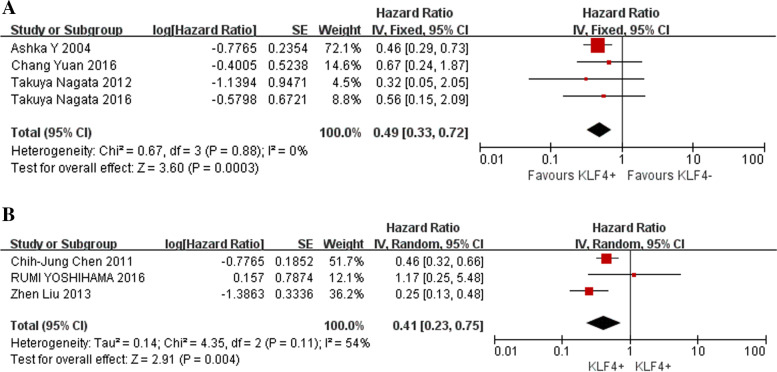
Forest plot and pooled HR and 95% CI for OS of breast cancer (BC) (**A**) and head and neck cancer (**B**)

In other cancers, high expression of KLF4 was associated with increased OS in BC (HR =0.49, 95% CI: 0.33–0.72) (Fig. 5A) and head and neck tumours (HR =0.41, 95% CI: 0.23–0.75) (Fig. 5B).

## Discussion

The cell cycle regulates the proliferation and differentiation of tumour cells, and various transcription factors affect the progression of the cell cycle by activating or transcribing cell cycle-regulating genes. KLF4 is a transcription factor that is widely present in eukaryotic cells with zinc finger structures and can promote cancer or tumour suppressors in different types of tumours. A large number of researchers have studied the expression of KLF4 in different malignancies. In numerous clinical studies, overexpression of KLF4 inhibited cell growth, migration, invasion and metastasis in HCC, lung cancer and CRC and induced tumour cell apoptosis in oesophageal cancer and bladder cancer [6, 46]. These functional investigations suggest that decreased expression of KLF4 promoted tumour progression and poor prognosis.

A meta-analysis of 17 studies on malignant tumours of the digestive system showed that a decrease in KLF4 was associated with poorer survival outcomes [47]. The underlying mechanism of KLF4 downregulation in cancer remains to be elucidated. There are several mechanisms of KLF4 as an oncogene: (i) inhibitor of Wnt/beta – catenin pathway: Yu [48] has found that KLF4 could be combined with the *β*-catenin, and then inhibited beta-catenin getting into the nucleus and binding with T cell factor, leading to the suppression of the downstream target gene of Wnt/beta-catenin pathway; (ii) regulation of the Notch pathway to inhibit tumour development: Ghaleb [49] found that Klf4 was the downstream target gene of the Notch pathway, and its transcription activity was inhibited by Notch. In the tumour, the Notch gene was highly expressed, and KLF4 was downregulated by the C-side control element of Notch (ICN1); (iii) KLF4 plays a role in epithelial-to-mesenchymal transition (EMT): KLF4 can inhibit EMT through regulation of E-cadherin gene expression [50]; (iv) immune escape mechanism: tumour cell major histocompatibility complex (MHC) changes, affecting antigen presentation, thus avoiding immune surveillance. KLF4 inhibits the expression of MHC and thus inhibits tumour development [51]. (v) Inhibitor of cell cycle KLF4 cooperates with p53 to enhance p21 expression and inhibit the function of cyclin D1 and cyclin B1, which causes cell cycle arrest at the G1/S and/or G2/M checkpoints [52].

KLF4, in skin cancer and BC, appears to promote tumour progression, suggesting that KLF4 is an oncogene in these tumours [10, 12]. The mechanism of KLF4 as an oncogene is vague, but Hu’s study [53] found that the oestrogen receptor (ER) can affect the E3 ubiquitin ligase, causing the degradation of KLF4 and accumulation of KLF4 protein. On the other hand, KLF4, as an agonist of ER transcription factors, can promote the ER and its downstream target gene promoter region and promote cell growth and mitosis. In addition, when DNA is damaged, KLF4 plays an anti-apoptotic effect by inhibiting the expression of p53 [54]. The deletion of KLF4 in BC can lead to the recovery of p53 expression and the apoptosis of p53-dependent tumour cells [12, 55]. This also confirms that KLF4 in BC is used as a cancer gene to promote cancer by inhibiting the expression of p53.

Our results show that KLF4 expression is not related to the prognosis of various malignancies. There was no difference between Asian cancer patients and non-Asian patients (HR = 1.44, 95% CI: 0.82–2.53, *P* = 0.2). Our study indicated that KLF4 might be a new biomarker for GC with a HR of 0.64 (95% CI: 0.49–0.84, *P* = 0.001). Nevertheless, 2 Asian studies on GC showed that high expression of KLF4 was associated with better prognosis, which was opposite to the American study. The reason was considered to be the sample size or race. However, further studies are needed to support this point. In subgroup analyses, high expression of KLF4 in the IHC group showed a better prognosis (HR = 0.72, 95% CI: 0.55–0.94, *P* = 0.2), whereas the RT-PCR group did not. This is probably because the expression of KLF4 is regulated at both the transcriptional and posttranscriptional levels. High expression of KLF4 was associated with good prognosis in BC (HR_OS_ = 0.49, 95% CI: 0.33–0.72) and head and neck tumours (HR_OS_ = 0.41, 95% CI: 0.23–0.75), which is different from previous results.

There are limitations to this study. First, the HRs of most studies, extrapolated based on Tierney’s method, were less reliable than those directly provided in the original articles. Second, studies published in other languages were not included, which probably introduced bias. Third, different tumours and different treatments were included in our study, which could result in great heterogeneity. Fourth, the cut-off values and detection methods in the studies were not uniform, which was also a source of heterogeneity. Fifth, because of the limited number of included studies of each type of cancer, the results of some carcinomas were statistically insignificant and might be less powerful. Finally, KLF4 expression could be different depending on the TNM stage, whereas stratification analysis by TNM staging was not performed since the original data could not be obtained. Although we used a random-effects model and conducted subgroup analyses to explore the potential source of heterogeneity, there were still unexpected heterogeneities in those subgroups. Therefore, future research should focus on high-quality studies with comprehensive evaluation, thus resulting in more standardized research and more accurate conclusions.

## Conclusion

Although our study showed that the expression of KLF4 is not related to the prognosis of the tumours included in the study, we found that high expression of KLF4 predicted a trend of better prognosis. Moreover, high expression of KLF4 is associated with prolonged OS in GC, BC and head and neck cancer and may be a predictive factor for these cancers. Numerous studies are needed to find out the role of KLF4, and the molecular basis of the transformation between tumour suppressor genes and oncogenes still needs to be solved.

## Data Availability

All data generated or analyzed during this study are included in this published article.

## Abbreviations

KLF4: Kruppel-like factor 4
HR: Hazard ratio
CI: Confidence interval
TNM: Tumor–Node–Metastasis
IHC: Immunohistochemistry
RT-PCR: Quantitative reverse transcription PCR
OS: Overall survival
DFS: Disease-free survival
RFS: Recurrence-free survival
PFS: Progression free survival
DSS: Disease-special survival
HCC: Hepatocellular carcinoma
CRC: Colorectal cancer
BC: Breast cancer
HNC: Head and neck tumor
GC: Gastric cancer
ESCC: Esophageal squamous cell carcinomas
PDAC: Pancreatic ductal adenocarcinoma
NHL: Non-Hodgkin’s lymphoma
CSCC: Cervical squamous cell carcinoma
MFS: Metastasis-free survival
ICN1: C-side control element of Notch
EMT: Epithelial-to-mesenchymal transition
MHC: Major histocompatibility complex
ER: Estrogen receptor
